# Supplementary material for: Maintaining resting cardiac fibroblasts in vitro by disrupting mechanotransduction
Source: PLoS One. 2020 Oct 26;15(10):e0241390. doi: 10.1371/journal.pone.0241390 (PMC7588109; doi:10.1371/journal.pone.0241390)
Supplement: Attachment — Submitted filename: Response to Reviewer.docx [file pone.0241390.s001.docx]

**Response from the Authors to the Reviewer comments for manuscript PONE-D-20-17115**

We thank the Editor and Reviewer for their helpful comments, thorough review and the invitation to submit a revised manuscript. We have addressed all specific comments below and marked the changes we have made to the manuscript (track changes) and figure legends (red text).

Please note that we have performed new experiments to address the Reviewer’s comments. These data are presented in the points below and in a new Figure 1.

We respectfully hope that you will find this paper suitable for publication in PLOS ONE.

*Reviewer #1: To understand the effect of mechanotransduction on fibroblasts to myofibroblasts differentiation of cardiac fibroblasts, it is highly critical to maintain the resting fibroblasts that are freshly isolated from tissues. The authors screened in vitro culturing conditions to slow down activation/differentiation of cardiac fibroblasts to myofibroblasts and recommend adding inhibitors specific to TGFβR1 and ROCK as well as seeding fibroblasts on soft hydrogels.
It is an important study, however, the manuscript needs to be revised thoroughly to address the following concerns.*

*Major issues
1. It is well established that TGFβR1 and ROCK play critical roles in fibroblasts to myofibroblasts differentiation and inhibitors of these signaling pathways inhibit the differentiation. The results described in this manuscript do not add anything substantially new to our current understandings.*

Although it is known that TGFβRI and ROCK are important for myofibroblast differentiation, their relative contributions and the effect of dual inhibition have not previously been tested. Our results bring novel information regarding the relative importance of these specific signaling pathways in regulating different myofibroblast-associated genes. E.g. the finding that col3a1 was upregulated by dual inhibition was a novel finding of this paper. Furthermore, combining blocker treatment with the use of soft hydrogels has not been tested previously and revealed that only some genes remain mechano-sensitive after phenotypic reversion. This is important information for researchers using inhibitors to maintain a “resting” cardiac fibroblast phenotype *in vitro* as well as for our current understanding of mechanosensitive gene regulation.

*It seems that once fibroblasts are activated through mechanotransduction by ECM stiffening to form SMA fibers, SMA fibers persist even after transferring cells onto a soft hydrogel or treatment with inhibitors of TGFβR1 and ROCK, therefore changes in cell morphology are negligible. LOX and LOX-family of proteins are upregualted via TGFβR1 signaling pathways, therefore upregulation of LOX gene was detected when TGFβR1 blocker was removed in Panel A and C (Figure 5). Authors need to cite literature concerning LOX gene and TGFβ.*

We thank the reviewer for suggesting this addition of information and respective citations. We have changed the text in the manuscript (page 11 line 22 and page 12 line 1-4) to:

“In contrast, Acta2, Lox and Postn were increased in cardiac fibroblasts on plastic and soft gels after blocker removal, suggesting that re-activation of TGFβRI and/or ROCK signaling is occurring regardless of matrix stiffness and are strong inducers of these genes (1, 2)”

We have cited the following articles:

1.	Voloshenyuk TG, Landesman ES, Khoutorova E, Hart AD, Gardner JD. Induction of cardiac fibroblast lysyl oxidase by TGF-beta1 requires PI3K/Akt, Smad3, and MAPK signaling. Cytokine. 2011;55(1):90-7.

2.	Lu M, Qin Q, Yao J, Sun L, Qin X. Induction of LOX by TGF-beta1/Smad/AP-1 signaling aggravates rat myocardial fibrosis and heart failure. IUBMB Life. 2019;71(11):1729-39.

*2. It is better to include a schematic diagram for relevant signaling pathways for fibroblasts to myofibroblasts differentiation.*

We would like to thank the reviewer for this excellent suggestion. In Figure 3A we have now added a schematic diagram of signaling pathways that induce myofibroblast differentiation in response to mechanical cues, and indicated the sites of inhibition by the tested blockers (Figure 3B). The figure legend and manuscript text were changed accordingly (page 8 line 19-22 and page 9 line 1).

*3. The authors need to provide rational/explanation for why they examine the effect of calcineurin inhibitor cyclosporine A in this study.*

We (3) and the Molkentin group (4) have previously shown that calcineurin-NFAT signaling is important for myofibroblast differentiation. We have now added this information to the introduction (page 4 line 11-12) to demonstrate the logic for investigating the effect of cyclosporine A.

*4. Images (Panel C and D) in Figure 3 (labeled as Supllemantary Figure 3 in the manuscript) should be replaced with images in better resolution.*

We apologize for the poor resolution in the submitted PDF. We have improved the resolution for the re-submission.

*5. It is intriguing that LOX is highly upregulated when cells are cultured on a plastic (Figure 1, Panel F and Figure5, Panel C).The authors need to add their explanation/interpretation for this.*

The stiffness of plastic is very high and will therefore lead to activation of several mechanosensitive signaling pathways (5). TGFβ signaling is such a mechanosensitive signaling pathways and has been shown to regulate Lox gene expression (1, 2). TGFβ is secreted in a latent complex that can be stored in the ECM. It is released by this latent complex in response to mechanical stress through binding to integrins on the cell surface. Since latent TGFβ will be present in the culture media (which is supplemented with 10% fetal bovine serum) and is produced by the cardiac fibroblasts themselves, it can become activated when cardiac fibroblasts are cultured on a stiff substrate such as plastic. The Hinz group have several excellent reviews describing this mechanism of mechanical activation of TGFβ (6-8).

To clarify the dynamics of Lox gene expression during culturing on plastic, we have performed experiments measuring Lox mRNA at different time points after cell isolation. These results are presented in the new Figure 1 in panel E and described in the revised manuscript (page 7 line 8-10). These results show that Lox gene expression gradually increases and is significantly elevated after 5-12 days on plastic.

*6. Figure legends in this manuscript need to be revised thoroughly. At the current state, it is not self explanatory.*

We have revised the Figure legends thoroughly, and thank the reviewer for point this out.

*Figure 1.
- Which data of Panel C do have N= 3 and which data of Panel C have N= 8?*

We have added details regarding the number of samples in each group.

*- Which data (day) do correspond to Panel F? What is Y-axis in Panel F?*

The protocol used for the data in Panel F is shown in Panel D. Cells were cultured on plastic for 9 days where after they were transferred to soft hydrogels for 4 days, giving a total of 13 days in culture. We have revised the figure legend extensively to clarify this.

The Y-axis in Panel F is mRNA shown as 2^-ΔCt^ and presents mRNA levels for each gene after normalization to the housekeeping gene.

ΔCt is the Ct value of gene of the gene interest minus the Ct value of the housekeeping gene. Since one Ct value reflects a doubling in mRNA, Ct values are transformed to a linear scale by exponentiation. Since Ct levels are inversely proportional to the amount of target nucleic acid in the sample, ΔCt is a negative value.

The data presented in Panel C where first transformed using 2^-ΔCt^ and then presented as relative to the control group to clearly show the effect of soft gels for each gene. The presentation in Panel F gives additional information about the level of expression among different genes.

*- Panel A and C, it is better to include more data points between day 9 and day 15 so that the critical time point for differentiation can be defined.*

We have now stained for SMA in cells cultured for 12 days on soft gels. The micrographs show that some cells have clear SMA fibers while other do not, indicating that some cells are starting to differentiate at this time point. We describe these results in the manuscript (page 7 line 22 and page 8 line 1-2). Although we used the same microscope and camera settings as for the previous immunocytochemistry staining, we prefer not to add these new images to the time line in Figure 2B since the immunostainings were not performed in parallel to the other time points and therefore there might be slight differences in SMA staining intensity. Below is a representative micrograph of the 12 day time point and the no primary antibody negative control. The scale bar is 100µm.

SMA (green)

DAPI (blue

12 days on soft gel

No primary antibody

SMA staining

*- Panel D describes fibroblasts cultured 9 days on plastic and switched to soft hydrogel and cultured for additional 4 days, but this is not explained in the legend. Is it necessary to incubate 9 days to see full differentiation? Some explanation for this experimental set-up needs to be provided.*

We have revised and explained the protocol more closely in the figure legend.

To characterize the dynamics of myofibroblast differentiation on plastic we have measured Acta2, Col1a1 and Lox mRNA (new Figure 1A, D and E, respectively) after different culturing times on plastic. We also stained for SMA protein and quantified the staining intensity (new Figure 1B and 1C). Both Acta2 mRNA and SMA protein peaked after 9 days on plastic. At this time point Col1a1 and Lox mRNA were also high, suggesting full differentiation into myofibroblasts. Therefore, this is an appropriate time point to study the potential for reversing myofibroblasts by culturing them on soft gels.

The manuscript has been changed to describe these new data in a new paragraph on page 7 line 2-10. We would like to thank the reviewer for raising this excellent point. We believe the new experiments and figure has greatly improved the paper.

*- The text of this manuscript mentions Figures 2E and 2F but are actually Figures 1E and 1F, respectively.*

We thank the reviewer for pointing out this important typo. Since we have now added a new Figure 1, the subsequent figures have new numbering. We have changed the manuscript accordingly.

*Figure 2.
- The label “anti-SMA” in Panel A, B, C should be SMA.*

This has been changed.

*- What are the differences between Panel B 50 μM and Panel C 50 μM?*

It is only the presentation of the image that is different: Brightness was increased in Panel C to show autofluorescence and thereby visualize the morphology of the cells. This has now been specified in the figure legend.

*Supplementary Figure 3.
- This is called Supplementary Figure 2 within the text, but it should be Figure 3.*

We apologize for this mistake, and have corrected the figure numbers and names.

*- Panel A. “wth” should be “with”*

This has been corrected.

*- Panel C and D. Description of upper and lower panels for effect of blockers need to be provided.*

This has been corrected and the labels SMA and auto-fluorescence have been added to the figure.

*Figure 4.
- Panel A and B, provide detailed procedures, e.g. Fibroblasts were cultured on plastic for 6 days (Panel A) and 13 days (Panel B).*

We have added this information to the figure legend.

*- Panel C and D should include relative mRNA levels of these markers before addition of SB+Y27.*

We have added the bar for the control group that was not treated with blockers. Since the 9 day and 16 day reverse experiments (Figure 5C and D) were separate cell isolations, we normalized to the respective non-treated control for each time point. The non-normalized data are shown below. The aim of the experiment in Figure 5 was to test the effect of SB+Y27 on gene expression. In our opinion, the relative values demonstrate this effect more clearly.

*Other issues
1. There are too many non-essential and introductory elements in a sentence and that makes this manuscript difficult to follow.*

We have reduced or removed introductory elements on page 7 line 14-15, page 8 line 10-12, page 8 line 19, page 9 line 10-13, page 10 line 7-8,

*2. Abstract
The last sentence, starting with Importantly, needs to be revised to address the conclusion of this paper and the message should be clearer.*

We have changed this sentence to “However, actin alpha 2 smooth muscle (Acta2), lysyl oxidase (Lox) and periostin (Postn) were no longer sensitive to substrate stiffness, suggesting that transient treatment with mechanotransduction inhibitors changes the mechanosensitivity of some fibrosis-related genes” (page 2 line 17-18)

*3. Introduction
Line 6 from the top, lysyl oxidase-like 2 (LOXL2) should be added as LOXL2 has also shown to play critical role in cardiac interstitial fibrosis by Yang, J. et al Nature Comm. 2016, 7, 13710.*

The paper by Yang and colleagues is most interesting and we thank the reviewer for drawing our attention to it. We have added Loxl2 to the list of proteins important for cardiac fibrosis and cite Yang accordingly (page 3 line 7-8).

*4. Results
The following sentences need to be revised for better understanding.
Relieving cells of high stiffness delays, but does not prevent myofibroblast differentiation.*

We have changed this sentence to “Culturing cardiac fibroblasts on soft gels delays myofibroblast differentiation” to improve understanding (page 7 line 12-13).

*Line 2 in the second paragraph, “SMA staining intensity was low for cardiac fibroblasts cultured on soft gels for 3 and 9 days, but increased after 15 days on gels (Figure 1B), at which time cells were highly confluent”*

We have changed this sentence to “SMA staining intensity was weak in cardiac fibroblasts cultured on soft gels for 3 and 9 days, but strong after 15 days on gels (Figure 1B)” to improve understanding (page 7 line 21-22).

*◊ SMA staining and confluency between day 9 and day 15 should be included (e.g. day 11, day 13).*

We have stained for SMA in cardiac fibroblasts cultured on soft gels for 12 days. Please see micrographs and our previous response to Reviewer on page 4.

*Line 6 in the second paragraph,
“Marker expression was no longer suppressed by soft compared with stiff culture substrates.”*

We have changed this sentence to “…marker expression was no longer reduced in cardiac fibroblasts on soft gels compared to plastic …” (page 8 line 6-7).

*“for the reverse experiment, SB inhibition, as well as all combinations of blockers, reduced SMA staining intensity, albeit the effect of blocker combinations were more pronounced.”*

We have changed this sentence to “For the ***reverse*** experiment, SB reduced SMA staining intensity, and this effect was augmented by adding Y27 or CsA” (Page 10 line 1-3).

*Minor
1. Full name and abbreviations.
Abstract:
Line 8 from the top, (SMA) should be inserted after smooth muscle α-actin.*

Corrected

*Line 10 from the top, transforming growth factor β receptor I should be inserted before (TGFβRI) as well as Rho-associated protein kinase before (ROCK). Actin alpha2 smooth muscle gene should be inserted before (Acta2).*

Corrected

*Introduction:
Line 4 from the top, full name for Acta 2 should be given.*

Corrected

*Line 9 from the top, full name for Tcf21 should be given.*

Corrected

*2. Some errors listed below need to be revised.
Abstract:
Line 4 from the top, “It is therefore challenging to study resting cardiac fibroblasts and their activation, in vitro.” The comma should be removed.*

Corrected

*Line 6 from the top, “maintain resting cardiac fibroblasts for up to 5 days but some experiments” Insert comma before but*

Corrected

*Introduction:
line 5 from the bottom, “However, the use of soft hydrogels have limitations” ◊ “The use of soft hydrogels has some limitations”*

Corrected

*Results:
Under “Inhibiting ROCK and TGFβRI is most efficient for preventing and reversing the myofibroblast phenotype” on the second page,
In the paragraph starting with “For the reverse experiment, “
“blocker combinations where most efficient in preventing and reversing” should read as “blocker combinations were most efficient at preventing and reversing”*

Corrected

*in the paragraph starting with “We next measured…”
“only SB prevented Acta2 upregulation confirming that” Insert a comma after upregulation.*

Corrected

1.	Voloshenyuk TG, Landesman ES, Khoutorova E, Hart AD, Gardner JD. Induction of cardiac fibroblast lysyl oxidase by TGF-beta1 requires PI3K/Akt, Smad3, and MAPK signaling. Cytokine. 2011;55(1):90-7.

2.	Lu M, Qin Q, Yao J, Sun L, Qin X. Induction of LOX by TGF-beta1/Smad/AP-1 signaling aggravates rat myocardial fibrosis and heart failure. IUBMB Life. 2019;71(11):1729-39.

3.	Herum KM, Lunde IG, Skrbic B, Florholmen G, Behmen D, Sjaastad I, et al. Syndecan-4 signaling via NFAT regulates extracellular matrix production and cardiac myofibroblast differentiation in response to mechanical stress. J Mol Cell Cardiol. 2013;54:73-81.

4.	Davis J, Burr AR, Davis GF, Birnbaumer L, Molkentin JD. A TRPC6-dependent pathway for myofibroblast transdifferentiation and wound healing in vivo. Dev Cell. 2012;23(4):705-15.

5.	Herum KM, Lunde IG, McCulloch AD, Christensen G. The Soft- and Hard-Heartedness of Cardiac Fibroblasts: Mechanotransduction Signaling Pathways in Fibrosis of the Heart. J Clin Med. 2017;6(5).

6.	Hinz B. The extracellular matrix and transforming growth factor-beta1: Tale of a strained relationship. Matrix Biol. 2015;47:54-65.

7.	Hinz B. It has to be the alphav: myofibroblast integrins activate latent TGF-beta1. Nat Med. 2013;19(12):1567-8.

8.	van Putten S, Shafieyan Y, Hinz B. Mechanical control of cardiac myofibroblasts. J Mol Cell Cardiol. 2016;93:133-42.
