# Supplementary material for: Maintaining resting cardiac fibroblasts in vitro by disrupting mechanotransduction
Source: PLoS One. 2020 Oct 26;15(10):e0241390. doi: 10.1371/journal.pone.0241390 (PMC7588109; doi:10.1371/journal.pone.0241390)
Supplement: Attachment — Submitted filename: Response to Reviewers.docx [file pone.0241390.s002.docx]

**Response to the Reviewer comments for manuscript PONE-D-20-17115**

We thank the Editor and Reviewer for their positive response. We have addressed all specific comments below and in the revised manuscript (track changes).

*Reviewer #1: The authors made efforts to address my concerns and the manuscript reads significantly better.
However, there are some issues that need to be sorted out before considering this manuscript for publication on this journal.

Major problem:
I am not sure why images are still at low resolution in this revision, although authors stated that they replaced older images with images in higher resolution. Perhaps it is due to how this PDF version of article was generated. Figures and legends are fuzzy with some background. Immunostaining images are not in publishable quality.*

We refer to the Editor’s comment on this point: “I understand that the reviewer probably did not find the high resolution figures available by clicking the link located at the upper right corner of each figure on the PLoS website, so your figures are fine as is with respect to resolution.”

*1. The authors response to the first critique under Major issues, “It is well established that TGFβR1 and ROCK play critical roles in fibroblasts to myofibroblasts differentiation and inhibitors of these signaling pathways inhibit the differentiation. The results described in this manuscript do not add anything substantially new to our current understandings.” is reasonable.

“Although it is known that TGFβRI and ROCK are important for myofibroblast differentiation,
their relative contributions and the effect of dual inhibition have not previously been tested.”
- This provides the rational for the research conducted in this study and should be included in the introduction.*

We have included this information in the Introduction page 5 line 15-17.

*“Our results bring novel information regarding the relative importance of these specific signaling
pathways in regulating different myofibroblast-associated genes. E.g. the finding that col3a1 was upregulated by dual inhibition was a novel finding of this paper. Furthermore, combining blocker treatment with the use of soft hydrogels has not been tested previously and revealed that only some genes remain mechano-sensitive after phenotypic reversion. This is important information for researchers using inhibitors to maintain a “resting” cardiac fibroblast phenotype in vitro as well as for our current understanding of mechanosensitive gene regulation.”
- This part should be included in abstract and discussion/conclusion.*

We have included this information in the Discussion on page 14 line 9-12 and in the Abstract page 3 line 1-7.

*2. Abstract Line 11-12 “Myofibroblast differentiation was prevented after 3 and 9 days on soft hydrogels. However, after 15 days, myofibroblasts appeared.”
“after 3 and 9 days on soft hydrogels” à”at 3 and 9 days after transferring to soft hydrogels”*

We apologize, but we are uncertain what the Reviewer is suggesting in this point.

However, we clarified this sentence to read “Myofibroblast differentiation was prevented on soft hydrogels for 9 days, but had occurred after 15 days on hydrogels. Transferring myofibroblasts to soft hydrogels reduced expression of myofibroblast-associated genes, albeit SMA fibers remained present.” on page 2 line 11-15, to include our results of transferring myofibroblasts to soft hydrogels.

We hope this addresses the concern of the Reviewer.

*3. Figure 2
Figure legend should read, “mRNA expression of myofibroblast markers and Tcf21 at 13 days after seeding. Y-axis value, 2-∆Ct, represents mRNA levels for each gene relative to 18S rRNA. ∆Ct is the Ct value of the gene of interest minus the Ct value of 18S rRNA.”*

This has been included (page 18 line 9-14).

*The following explanation should be included in text. “One Ct value reflects a doubling of mRNA and Ct values are transformed to a linear scale by exponentiation. Since Ct levels are inversely proportional to the amount of target nucleic acid in the sample, ∆Ct is a negative value. The data presented in Panel C where first transformed using 2-∆Ct and then presented as relative to the control group to clearly show the effect of soft gels for each gene.”*

We have included the following in the Methods page 7 line 9-13: “Since one Ct value reflects a doubling of mRNA, Ct values were transformed to a linear scale by exponentiation. Ct levels are inversely proportional to the amount of target nucleic acid in the sample; therefore, ∆Ct is a negative value. The data presented in Figure 2F were presented as 2^-∆Ct^. All other mRNA data were presented as relative to the control group.”

*The difference between Figure 2C (little effect between soft gel and plastic) and Figure 2F (very high difference after transferring to soft gel) is striking. Could this be related to confluency of cells? It seems unlikely that cells transferred to soft gels kept the exact same confluency as those kept on plastic.*

Indeed, the high confluency after 15 days on gels could obscure the effect of soft hydrogels as discussed in the second paragraph of the Discussion (page 13 line 6-12). However, there are no obvious differences in confluency between the cells on plastic and the cells transferred to gels in Figure 2E. Thus, the pronounced effect of transferring myofibroblasts to soft hydrogels, indicates that once cardiac fibroblasts have differentiated into myofibroblasts, a stiff substrate is necessary for continued expression of myofibroblast-associated genes. This point has been added to the Results section page 9 line 10-12.

*4. Figure 4
Panels A and B should have days on the arrows similar to Figure 2.*

The red arrows in Figure 2 indicate addition of fresh blocker solution every 24h for three days, whereas in Figure 2 the arrows indicate the time when cells were harvested for analysis. Thus, we believe it might cause confusion to label the arrows similarly in both figures. Instead, we have added the following explanation to the figure legend of Figure 4: “Red arrows indicate the addition of fresh inhibitor solution every 24h” (Page 19, line 11-12)

*5. Figure 5
Figure legend for panel B is missing.*

We have now added the legend for panel B (page 20 line 2-3) and thank the reviewer for pointing out this lack of information.

*The authors need to include some explanation/discussion about the difference in the level of Col3a1. Why it is 2X increased for reversed in panel C but no effect in D?*

We believe that the long initial culturing time on plastic in panel D renders the cells less responsive to inhibitors of mechanotransduction. Thus, fewer genes were affected by TGFβRI and ROCK inhibition in panel D. We have added the following sentence (page 11 line 20-21 and page 12 line 1-2) to specifically address the observed effects on Col3a1 expression: “Furthermore, the induction of Col3a1 expression in response to inhibitor treatment only occurred at the early time point, indicating that long culture times on plastic render cardiac fibroblasts more resistant to these pharmacological inhibitors.”
